# Different Statistical Approaches to Develop a Guideline for Improvement of Caregiver’s Mental Health

**DOI:** 10.1093/geroni/igab046.3109

**Published:** 2021-12-17

**Authors:** Amanda Leggett, Hyun Jung Koo

**Affiliations:** 1 The University of Michigan, Ypsilanti, Michigan, United States; 2 University of Michigan, Ann Arbor, Michigan, United States

## Abstract

Caregiver burden is common, and improvement of caregivers’ mental health could lead to better quality of care and well-being for both caregivers and care recipients. We investigate ways to develop a guideline to enhance caregiver’s mental well-being by applying and comparing regression tree and ensemble tree models. Data comes from the 2017 National Health and Aging Trends Study and National Study of Caregiving. Dementia caregivers’ (n=945) aspects of caregiving, care activities, support environment, and participation along with basic demographics and health are considered. First, insignificant predictors are preselected using linear regression with backward selection, which will not be included in the tree models. Using the predetermined predictors that are not excluded in the backward selection method, regression tree and ensemble tree models are generated to predict emotional difficulty of caregivers. The regression tree with the preselected predictors predicts caregivers with low to moderate levels of overload and high levels of joy being with their care recipient associated with the lowest level of emotional difficulty. On the other hand, if caregivers have high levels of overload and low to moderately high levels of positive affect, this is linked with the highest level of emotional difficulty. Ensemble tree models showed similar results with lower error measures. Using tree-based methods can help determine the most important predictors of caregiver mental health. Easily interpretable results with applicable decision rules can provide a guideline for intervention developers.

